# Serotonin Reuptake Inhibitors in Pregnancy: Can Genes Help Us in Predicting Neonatal Adverse Outcome?

**DOI:** 10.1155/2014/276918

**Published:** 2014-01-12

**Authors:** Valentina Giudici, Laura Pogliani, Dario Cattaneo, Dario Dilillo, Gian Vincenzo Zuccotti

**Affiliations:** ^1^Department of Paediatrics, Hospital Luigi Sacco, Via GB Grassi 74, 20157 Milan, Italy; ^2^Unit of Clinical Pharmacology, Hospital Luigi Sacco, Via GB Grassi 74, 20157 Milan, Italy

## Abstract

Lots has been written on use of SSRI during pregnancy and possible short and long term negative outcomes on neonates. the literature so far has described a various field of peripartum illness related to SSRI exposure during foetal life, such as increased incidence of low birth weight, respiratory distress, persistent pulmonary hypertension, poor feeding, and neurobehavioural disease. We know that different degrees of outcomes are possible, and not all the newborns exposed to SSRIs during pregnancy definitely will develop a negative outcome. So far, still little is known about the possible etiologic mechanism that could not only explain the adverse neonatal effects but also the degree of clinical involvement and presentation in the early period after birth. Pharmacogenetics and moreover pharmacogenomics, the study of specific genetic variations and their effect on drug response, are not widespread. This review describes possible relationship between SSRIs pharmacogenetics and different neonatal outcomes and summarizes the current pharmacogenetic inquiries in relation to maternal-foetal environment.

## 1. Introduction

Selective serotonin reuptake inhibitors (SSRIs) are commonly used antidepressants that act by inhibiting serotonin reuptake in the synaptic cleft. Medications in this group include fluoxetine, paroxetine, sertraline, fluvoxamine, citalopram, and escitalopram. At higher doses, paroxetine and sertraline also block dopamine reuptake, which may contribute to their antidepressant action. Venlafaxine is a combined serotonin-norepinephrine reuptake inhibitor. Recently, multiple studies have correlated SSRI use during pregnancy with adverse neonatal effects, including neonatal respiratory distress, persistent pulmonary hypertension, jaundice, feeding problems, abnormal movements and tonus abnormalities, birth weight below 10° centile, and even congenital cardiac disease (paroxetine only). We know very well from the literature that approximately 15% of all pregnant women have psychiatric problems, in particular depression and anxiety [1, 2]; we also know that the possible negative effects of untreated psychiatric symptoms have to be compared and weighted against the possible negative effects of medication during pregnancy both in mother and foetus. The decision to treat psychiatric disorders during pregnancy needs to be evaluated in every single case and the literature has provided us with many guidelines and reviews which can help in the daily clinical practice [3–5] However, these position papers mainly focus on the risk of foetal malformation and little is provided regarding the early neonatal outcomes of which every neonatologist and pediatrician should be aware of. As we understand the adverse foetal effects of specific psychotropic medications, it is clear that not all fetuses exposed to a given medication will show evidence of its associated possible negative outcomes and that these effects may be varied in both their presentation and timing. Therefore, the capacity to identify foetal risk in an increasingly sensitive manner would greatly benefit the specificity with which we will be able, as physicians, to deal with possible negative neonatal outcomes.

This paper not only focuses on early neonatal outcome after foetal exposure to a serotonin reuptake inhibitors drug but also tries to investigate both the possible etiology of what is commonly called the poor neonatal adaptation and in particular what are the possible predictors of adverse outcome.

Our aim was to evaluate the current literature on this topic, moreover investigating the possible correlation among genes polymorphisms and different outcome in newborn exposed to SSRI during foetal life. To the best of our knowledge only a few paper have focused on this topic.

## 2. Methods

We searched PubMed (2003–2013) using the key words SSRI pharmacogenetics, newborn, and SSRI pharmacokinetics, neonatal withdrawal syndrome combining them in couple or triplets, producing several different search strategies. We searched also for neonatal pharmacogenomics and pregnancy. Papers related to early neonatal outcomes and pharmacogenetics of SSRI were acquired. More specifically we aimed to evaluate the state of art so far regarding genotypic correlation to adverse neonatal outcome in newborn exposed to SSRI during foetal life. Inclusion criteria were identified maternal SSRI exposure during pregnancy, presence of neonatal outcome assessment, and evaluation of molecular/genotypic influences on outcome. This search produced more than 1500 publications, subsequently included publications were limited to meta-analyses, randomized controlled trials, clinical trials, practice guidelines, reviews and case report and in total 108 publications were selected. Among these we excluded 79 publications because they did not meet our criteria or because they did not include a significative population sample. Finally we included in our review 36 publications. In our reference list we included other publications which we thought to be of interest in the setting of the topic debated.

Publications were excluded if

- non-English literature;
- the publication did not address infant born of mothers who had been treated with SSRI during pregnancy;
- the publication did not address neonatal adverse effects and among them were excluded publication focused on malformative complications;
- the publication did only address SSRI effects on pregnant woman regardless of the newborn.

## 3. Discussion

Symptoms of poor neonatal adaptation are clearly related to psychotropic drugs exposure during the whole trimesters of pregnancy, but still the exact etiology is not fully understood. A recent paper from Kievet et al. [6] stated three possible explanation beyond the negative outcome in a newborn from a depressed and on psychotropic treatment mother: symptoms in newborn could be essentially caused by withdrawal syndrome, since the psychotropic drugs will pass through the placenta and therefore abrupt discontinuation could lead to a spectrum of symptoms which easily mimic the adult withdrawal syndrome. Another cause could be drug toxicity, in which case symptoms may develop soon after birth, due to drug plasma concentration higher than the ones observed in newborn with withdrawal syndrome. Third, it has been hypothesized that all psychotropic drugs may act as intrauterine stressors and therefore they could start foetal alteration in development, which could lead to child's behavior alteration at any age after birth. In withdrawal syndrome symptoms occur soon after birth in a range of time that goes approximately from 8 to 48 hours after delivery; the most typical symptoms include feeding difficulties, irritability, and tremors [7]. Sometimes newborn can even present with sleeping difficulties, vomiting, and hypo- or hypertonia. Respiratory distress can be common, as previously documented its incidence can be significantly higher in SSRI exposed neonates (13.9%) than the other neonates [8]. Symptoms of drug toxicity can be partly similar to symptoms of withdrawal, such as tremors and hypertonia. However, the main difference is that in this condition symptoms occur immediately after birth, as in most cases plasma concentration of psychotropic drugs are higher. Newborn can present with irritability as well, and often hyperreflexia and respiratory distress can be part of the spectrum. Since it can be difficult to clearly distinguish withdrawal from toxicity it has been hypothesized that a combination of both can be possible [9, 10], giving rise to a larger spectrum of clinical presentation consisting of neurological, autonomic, gastrointestinal, and respiratory symptoms. It is of extreme importance to note that some newborn experience a mild presentation, for example, with sleep disorders or gastrointestinal symptoms. Some others instead present with more severe symptoms such as neurological disorders, from tremors to convulsive episodes or even with important respiratory distress. Many studies reported that nearly 30% of all infants exposed to SSRI during foetal life present with poor neonatal adaptation [11, 12] and some of them described an increased incidence of neonatal symptoms after exposure to paroxetine and fluoxetine compared to other SSRIs [9, 13, 14]. On the basis of adult-based extrapolation of SRI pharmacology we know that the risk of withdrawal syndrome or toxicity seems to be related to the half-life of SSRIs: the class agents with a short half-life, such as paroxetine, are described to cause more withdrawal symptoms that occur with declining drug levels. Alternatively, exposure to long-life SSRIs like fluoxetine could be more likely associated with neonatal toxicity syndrome, with immediate onset of symptoms after birth [14]. Some pharmacokinetic data support this [15, 16]: concentration of fluoxetine seems to remain unchanged in infants between delivery and the first 48 hours of life, while concentrations of other SSRIs decrease even by 60% after the first 48 hours of life, and among them paroxetine seems to be metabolized most rapidly [17]. As already stated in the literature, mechanisms that clearly explain why only some infants who have been exposed to maternal use of SSRIs during foetal life present with poor adaptation after birth are still unknown. It is now well known that the serotonin transporter 5HTT plays an important role as a regulator of serotoninergic neurotransmission and modifier of SSRIs effects: differences in transporter-dependant reuptake efficiency are related to insertion/deletion polymorphism in the promoter region of the SLC6A4 gene, thus leading to different transporter gene expression and clinical differences in SSRI efficacy [18]. To the best of our knowledge so far only three papers have investigated the possible relationship between genotypes and possible adverse outcome, as shown in Table 1.

An interesting paper from Oberlander et al. [19] sought to investigate whether neonatal effects of SSRIs are related to genotypes for the serotonin transporter (SLC6A4) promoter; specifically, whether affected neonates were carriers of the short or long SLC6A4 alleles. The study suggested that prenatal SSRI exposure was associated with adverse neonatal outcomes and these effects were moderated by infant SLC6A4 genotypes. More specifically risk of respiratory symptoms was higher in neonates exposed to SSRI in utero who had the long genotypes, compared to nonexposed neonates. Moreover, the relationships between polymorphisms and specific outcomes varied during the neonatal period, suggesting that beyond apparent gene-medication interactions, multiple mechanisms contribute to the adverse neonatal outcomes after prenatal SSRI exposure; see Tables 2 and 3.

Prenatal serotonin reuptake inhibitor exposure is common and neonatal outcomes vary greatly, often leading to confusion about whether to use or even continue antenatal use of these antidepressants. Importantly, some but not all infants are affected, which raises questions about how maternal drug metabolism contributes to fetal drug exposure. To address this question, here we briefly review the role of maternal, fetal, and placental genetic factors that affect the extent of fetal drug exposure.

### 3.1. Genetic Factors Affecting Infant Exposure to SSRIs

An interesting study from Laine et al. [20] in 2004 reported a case of a newborn who experienced severe complications after birth and the severity of these symptoms was significantly associated with the low cord blood 5-hydroxyindoleacetic acid concentrations, a marker for serotonin turnover, but not with infant plasma drug levels, thus suggesting that the perinatal sequelae were related to high central nervous system serotonin activity rather than discontinuation syndrome. At the molecular analyses this newborn was found to be a carrier of two defective CYP2D6 alleles, resulting in inactive CYP2D6 enzyme. In this respect, the SSRI drug plasma level in newborn can be surprisingly low, even if the neonates presents with serotoninergic symptoms which can easily mimic a withdrawal syndrome—Table 4; despite the low plasma concentrations of SSRI drug, the absence of CYP2D6 in the central nervous system could have caused high drug concentrations at the effector site, leading to strong serotonin reuptake inhibition and serotoninergic overstimulation in the infant; therefore, from a clinical-practical point of view it is once more important to keep in mind that the so-called serotoninergic symptoms are sometimes indistinguishable from SSRI withdrawal.

Similarly to what happens with maternal opiate exposure, signs of intoxication can present immediately and dissipate over a matter of hours, whereas withdrawal may take days or weeks to resolve. This is indirectly supported by opposite findings describing a case of a baby born from parents categorized as extensive metabolizers according to their CYP2D6 genotype who experienced an episode of SSRI withdrawal reaction, although he was not at an increased genetic risk of drug accumulation. Taken together, these cases suggest that withdrawal may account for the abnormal clinical findings in these patients. Additionally, as underlined by Stiskal [21] the differential diagnosis between SSRI neonatal withdrawal and serotoninergic symptoms is not merely theoretical. Indeed a withdrawal should be optimally treated with SSRI, whereas such treatment may endanger babies exhibiting serotoninergic syndrome.

Further evidence regarding the importance of genotypic pattern was added in 2009 with a controlled prospective study from Hilli et al. [22] who were the first to investigate the relationship between relevant genetic polymorphisms and monoamine concentrations in newborn who were exposed to SSRI during late pregnancy; they found that rapid MAO-A metabolizers had higher scores for perinatal serotoninergic symptom and therefore suggested that MAO-A genotype could be an independent risk factor for occurrence and severity of perinatal serotoninergic symptoms after pregnancy exposure to SSRI.

Recent studies have illustrated the importance of placental drug transport proteins, such as P-glycoprotein (Pgp) and breast cancer resistance protein (BCRP) in limiting fetal exposure to drugs and toxins. Moreover, increasing evidence supports a role for Pgp and BCRP in the normal development and physiological function of the placenta. Several single nucleotide polymorphisms (SNPs) in the genes encoding Pgp and BCRP have been described and are associated with altered protein expression, transporter activity, and clinical outcome in studies focusing on tissues other than the placenta [23]. To date, evidence suggests that SNPs in both ABCB1 and ABCG1 can alter expression of their respective protein; however, the functional significance of these polymorphisms in regulating the placental transfer of SSRI is not clear. Indeed, consistent evidence is available showing that polymorphisms in ABC drug efflux transporters significantly affected SSRI exposure. However, studies specifically dealing with pregnancy outcome are lacking.

In more details to the best of our knowledge the first paper to describe a possible association between ABCB1 variants with SSRIs response was the interesting paper from Kato et al., [24] which examined the possible association of 3 functional ABCB1 polymorphisms (C3435T: rs1045642, G2677T/A: rs2032582, c1236t: RS1128503) with response to paroxetine in a Japanese major depression sample cohort. In their results they showed a significant association of the nonsynonymous single nucleotide polymorphism G2677T/A with a treatment response to paroxetine; furthermore, the wild variants haplotype resulted are associated with poor response.

On respect of this topic, another paper from de Klerk et al. [25] studied the possible association of ABCB1 gene variants with adverse effects of SSRIs which among the antidepressants show a major affinity as substrate for P-glycoprotein. In their cohort of 424 depressed patients they found a significant association between the number of SSRI-related adverse drug effects and two single variants and one haplotype of the gene. They concluded that the serotoninergic effects were significantly predicted by these variants and haplotype, therefore, two common polymorphisms of the ABCB1 gene.

### 3.2. Factors Affecting Infants Exposure to SSRI through Breast Milk

The treatment of breastfeeding mothers with depression raises several dilemmas, including the possible risk of drug exposure through breast milk for the infant against the disadvantage of not receiving mother's milk. [26]. As for SSRIs, evidence is available showing that detectable drug levels have been found in breast milk for all antidepressant studies. In general, drug concentrations in milk parallel those in maternal plasma, but with a slight delay.

Only a few and inconclusive studies have explored the role of genetic background on exposure of infants to SSRI though milk lactation. In their study of 25 breastfeeding mothers who were treated with citalopram, sertraline, paroxetine, fluoxetine, and venlafaxine, Berle et al. [27] evaluated multiple variations in maternal CYP2D6 and CYP2C19 genotypes, including genotypes associated with “poor metabolizing.” They found that no specific genotypes correlated with variations in excreted medications in breast milk and all of the infant's serum levels were either undetectable or low.

Taking all current knowledge into considerations it has been recently suggested that when antidepressant treatment is indicated in women with postpartum depression, they should generally not be advised to continue breastfeeding. If this is not pursuable, among the different SSRIs, paroxetine and sertraline are most likely suitable first-line agents due to their low excretion in milk. However, an individual risk-benefit assessment should always be performed.

### 3.3. Genetic Factors Affecting Maternal Exposure to SSRI during Pregnancy

Ververs et al. [28] examined the changes of maternal paroxetine concentrations during pregnancy in relation to cytochrome P450 (CYP) 2D6 genotype/phenotype. These authors reported that women who were genotyped as extensive metabolizers or ultrarapid metabolizers for CYP2D6 showed steadily decreasing plasma paroxetine concentrations during the course of pregnancy, with a decrease of 0.3 mg/L for each week of pregnancy. In contrast, plasma paroxetine concentrations of intermediate metabolizers and poor metabolizers increased during pregnancy, resulting in an increase of 0.82 mg/L for each week of pregnancy. Interestingly, in extensive/ultrarapid metabolizers the depressive symptoms increased significantly during the course of pregnancy, while in the intermediate/poor metabolizer groups these did not change. According to the latter findings, accumulation of paroxetine in a considerable group of pregnant women may lead to unintended increased exposure of paroxetine to the unborn child. Although not formally proven by ad hoc studies, it can be reasonably speculated that the same could apply also for other SSRIs, being mainly metabolized not only by CYP2D6 but also by CYP2C19, CYP3A, and CYP1A2 which in turn are encoded by highly polymorphic genes. Accordingly, it could be reasonably hypothesized that monitoring exposure of pregnant women to SSRI through pharmacogenetic approaches or through therapeutic monitoring of plasma SSRI concentrations may favor the early identifications of women at risk which could potentially beneficiate from drug dose adjustments [29].

The state of the art so far describes a small pattern of studies that are relevant in their potential impact of predicting possible adverse outcomes depending on genetic variants, and our wish is that further studies will be developed in the maternal-neonatal field, in order to implement the knowledge and possible clinical implications regarding foetal outcome and newborn health. A better understanding of these polymorphisms will allow prediction of when normal placental physiology could be altered and lead to the development of appropriate preventive therapies, in summary a full understanding of which SNPs affect placental expression and function would be useful for predicting altered foetal exposure to drugs.

## 4. Conclusion

Approximately 7–13% of infants born in the United States each year are exposed to maternal major depressive disorder during gestation and an estimated 7.6% of all pregnant women take an antidepressant. Because of their low side effect profiles and relatively low risk on the foetus, among antidepressants SSRIs are a current first-line choice for pharmacologic treatment during pregnancy, even due to the well-known fact that exposure to maternal depression in utero has been already linked to adverse neurobehavioural development.

We know that the placenta is the key regulatory organ which maintains foetal homeostasis; therefore, we strongly think that it is the critical site to examine if we want to early detect alterations in neurotransmitter or genetic variations which could potentially be involved in dysregulation of the intrauterine environment and therefore foetal development. These studies account for a strong clinical relevance when limiting foetal exposure to potential teratogens or when drug delivery to the foetus is warranted for therapeutic effects, as in the case of maternal depression.

When certain genetic variations can be linked with specific foetal/neonatal outcomes, individual gene sequencing may provide more knowledge for a safe maternal pharmacotherapies on an individualized basis and therefore contributing to better stratify the risk for possible adverse neonatal outcome. The development and application of this field could offer greater potential to better institute appropriate medical care but obviously raises some concerns about feasibility in daily clinical practice. Although research in the area of drug metabolism in pregnancy is rapidly evolving, physicians are often limited by lack of knowledge of pathophysiological mechanisms that link a specific drug and adverse outcomes, and we think that research in maternal-foetal-neonatal pharmacogenomics will undoubtedly not only improve our overall understanding but also help us to stratify patients based on individual risk.

Further studies must be considered, in order to better clarify the role of haplotypes, environmental factors, and comorbidities, both to avoid and predict possible clinical adverse outcomes on newborns from depressed mothers.

## Figures and Tables

**Table 1:** Principal papers which focused on the role of genotypes and possible neonatal outcomes.

Laine et al., 2004 [20]	32-year-old woman SSRI exposed	Chlorpromazine, paroxetine	Genetic analysis: CYP2D6*4 mutation in both alleles = poor metabolizer APGAR 7/8/9 Poorly reactive, respiratory distress, tremors, hypotonia, and hypoglycemia
Oberlander et al., 2008 [19]	37 women SSRI exposed versus 47 nonexposed	Paroxetine, fluoxetine, sertraline, venlafaxine, citalopram	SS alleles: lower 5 min APGAR score (reduced respiratory effort) LL alleles: lower birth weight
Hilli et al., 2009 [22]	20 women SSRI exposed during pregnancy and lactation	Citalopram Fluoxetine	No differences between 5HTT and 5HT receptors genotypes and serotoninergic symptoms scores. Higher scores in infants with two high activity alleles of the MAO-A polymorphism and rapid CYP2DS metabolizers.

**Table 2:** Genotypes as possible regulators of perinatal serotoninergic symptoms after in utero exposure to SSRIs—courtesy of Oberlander 2008—Molecular Psychiatry. Infant outcomes and SLC6A4 genotype: means (s.d.).

SLC6A4 genotype	ll		ls		ss	
	No exposure (*n* = 14)	SRI exposure (*n* = 14)	No exposure (*n* = 22)	SRI exposure (*n* = 16)	No exposure (*n* = 11)	SRI exposure (*n* = 7)
Duration of prenatal SRI exposure (days)	NA	240 (57)	NA	206 (92)	NA	231 (62)
Maternal mean daily dose of medication (*z* score^a^)	NA	0.181 (0.138)	NA	0.299 (0.283)	NA	0.153 (0.101)
Gestational age at birth (weeks)	39.8 (1.45)	39.4 (1.48)	40.3 (1.03)	39.3 (1.61)	40.3 (1.09)	39.4 (1.44)
Birth weight (g)	3583 (594)	3416 (509)	3691 (455)	3239 (549)*	3465 (545)	3763 (367)
Birth length (cm)	52.3 (3.80)	50.1 (2.27)	51.9 (2.70)	50.5 (2.42)	52.3 (2.40)	53.1 (2.28)
Head circumference (cm)	34.8 (1.49)	34.3 (1.34)	35.2 (1.28)	34.5 (1.17)	35.0 (1.14)	35.2 (1.68)
Length of newborn stay in hospitals (h)	51.0 (22.2)	61.3 (27.9)	48.6 (25.6)	61.3 (40.9)	46.9 (27.6)	62.1 (21.4)

NA: not applicable; SRI: serotonin reuptake inhibitors. *P* < 0.05 for differences between exposures and nonexposure within genotypes. ^a^A composite drug dosage “*z* score” was tabulated for drug dosage to account for multiple drugs each with varying drug dose ranges.

**Table 3:** Genotypes as possible regulators of perinatal serotoninergic symptoms after in utero exposure to SSRIs—courtesy of Oberlander 2008—Molecular Psychiatry. Frequency (%) of PNA symptoms by SLC6A4 genotype and SRI exposure.

SLC6A4 genotype	ll		ls		ss	
	No exposure (*n* = 14)	SRI exposure (*n* = 14)	No exposure (*n* = 22)	SRI exposure (*n* = 16)	No exposure (*n* = 11)	SRI exposure (*n* = 7)
Tachycardia (>160 bpm)	14.3	57.1	13.6	25.0	27.3	14.3
Bradycardia (<100 bpm)	7.1	0.0	4.5	0.0	0.0	0.0
Tachypnea breathing (>60 min)	14.3	57.1*	13.6	25.0	27.3	14.3
Respiratory distress	14.3	50.0*	9.1	43.8	9.1	42.9
Itteriness	0.0	35.7*	9.1	25.0	9.1	57.1*
Increased motor tone	0.0	14.3	0.0	0.0	0.0	42.9*
Hypoglycemia (<3.3 mmol/L)	7.1	21.4	9.1	25.0	9.1	14.3
Hyperglycemia (>7 mmol/L)	0.0	7.1	0.0	0.0	0.0	14.3

PNA: poor neonatal adaptation; SRI: serotonin reuptake inhibitors. *Compared with no exposure on Pearson's *χ* ^2^ (all *P* < 0.05).

**Table 4:** Neonatal symptoms possibly caused by SSRI withdrawal or toxicity.

Neurological	Tremor, hyper/hypotonicity/jitteriness, and convulsions
	Restlessness, apathy, abnormous cry, and abnormal sleep
Gastrointestinal	Diarrhoea, vomiting, and breast feeding problems
Autonomic	Temperature instability, increased sweating, and fever, and blood pressure and heart rate changes
Endocrine	Jaundice and hypoglycaemia
Respiratory	Respiratory distress, tachypnoea, and desaturation

## References

[1] Marcus SM, Flynn HA, Blow FC, Barry KL (2003). Depressive symptoms among pregnant women screened in obstetrics settings. *Journal of Women’s Health*.

[2] Andersson L, Sundström-Poromaa I, Bixo M, Wulff M, Bondestam K, Åström M (2003). Point prevalence of psychiatric disorders during the second trimester of pregnancy: a population-based study. *The American Journal of Obstetrics and Gynecology*.

[3] Yonkers KA, Wisner KL, Stewart DE (2009). The management of depression during pregnancy: a report from the American Psychiatric Association and the American College of Obstetricians and Gynecologists. *General Hospital Psychiatry*.

[4] National Institute for Clinical Excellence NICE antenatal and postnatal mental health.

[5] Bellantuono C, Migliarese G, Gentile S (2007). Serotonin reuptake inhibitors in pregnancy and the risk of major malformations: a systematic review. *Human Psychopharmacology*.

[6] Kievet N, Dolman K, Honig A (2013). The use of psychotropic medication during pregnancy: how about the newborn?. *Neuropsychiatric Disease and Treatment*.

[7] Boucher N, Bairam A, Beaulac-Baillargeon L (2008). A new look at the neonate’s clinical presentation after in utero exposure to antidepressants in late pregnancy. *Journal of Clinical Psychopharmacology*.

[8] Oberlander TF, Warburton W, Misri S, Aghajanian J, Hertzman C (2006). Neonatal outcomes after prenatal exposure to selective serotonin reuptake inhibitor antidepressants and maternal depression using population-based linked health data. *Archives of General Psychiatry*.

[9] Klinger G, Merlob P (2008). Selective serotonin reuptake inhibitor induced neonatal abstinence syndrome. *Israel Journal of Psychiatry and Related Sciences*.

[10] Hudak ML (2012). Tan RC neonatal drug withdrawal. *Pediatrics*.

[11] Sie SD, Wennink JMB, van Driel JJ (2012). Maternal use of SSRIs, SNRIs and NaSSAs: practical recommendations during pregnancy and lactation. *Archives of Disease in Childhood*.

[12] Oberlander TF, Misri S, Fitzgerald CE, Kostaras X, Rurak D, Riggs W (2004). Pharmacologic factors associated with transient neonatal symptoms following prenatal psychotropic medication exposure. *Journal of Clinical Psychiatry*.

[13] Sanz EJ, De-las-Cuevas C, Kiuru A, Bate A, Edwards R (2005). Selective serotonin reuptake inhibitors in pregnant women and neonatal withdrawal syndrome: a database analysis. *The Lancet*.

[14] Moses-Kolko EL, Bogen D, Perel J (2005). Neonatal signs after late in utero exposure to serotonin reuptake inhibitors: literature review and implications for clinical applications. *Journal of the American Medical Association*.

[15] Laine K, Heikkinen T, Ekblad U, Kero P (2003). Effects of exposure to selective serotonin reuptake inhibitors during pregnancy on serotonergic symptoms in newborns and cord blood monoamine and prolactin concentrations. *Archives of General Psychiatry*.

[16] Heikkinen T, Ekblad U, Palo P, Laine K (2003). Pharmacokinetics of fluoxetine and norfluoxetine in pregnancy and lactation. *Clinical Pharmacology and Therapeutics*.

[17] Costei AM, Kozer E, Ho T, Ito S, Koren G (2002). Perinatal outcome following third trimester exposure to paroxetine. *Archives of Pediatrics and Adolescent Medicine*.

[18] Pollock BG, Ferrell RE, Mulsant BH (2000). Allelic variation in the serotonin transporter promoter affects onset of paroxetine treatment response in late-life depression. *Neuropsychopharmacology*.

[19] Oberlander TF, Bonaguro RJ, Misri S, Papsdorf M, Ross CJD, Simpson EM (2008). Infant serotonin transporter (SLC6A4) promoter genotype is associated with adverse neonatal outcomes after prenatal exposure to serotonin reuptake inhibitor medications. *Molecular Psychiatry*.

[20] Laine K, Kytölä J, Bertilsson L (2004). Severe adverse effects in a newborn with two defective CYP2D6 alleles after exposure to paroxetine during late pregnancy. *Therapeutic Drug Monitoring*.

[21] Stiskal JA (2006). Defective alleles may not have contributed to adverse effects. *Therapeutic Drug Monitoring*.

[22] Hilli J, Heikkinen T, Rontu R (2009). MAO-A and COMT genotypes as possible regulators of perinatal serotonergic symptoms after in utero exposure to SSRIs. *European Neuropsychopharmacology*.

[23] Hutson JR, Koren G, Matthews SG (2010). Placental P-glycoprotein and breast cancer resistance protein: influence of polymorphisms on fetal drug exposure and physiology. *Placenta*.

[24] Kato M, Fukuda T, Serretti A (2008). ABCB1 (MDR1) gene polymorphisms are associated with the clinical response to paroxetine in patients with major depressive disorder. *Progress in Neuro-Psychopharmacology and Biological Psychiatry*.

[25] de Klerk OL, Nolte IM, Bet PM (2013). ABCB1 gene variants influence tolerance to selectiveserotonin reuptake inhibitors in a large sample of Dutch cases with majordepressive disorder. *The Pharmacogenomics Journal*.

[26] Berle JØ, Spigset O (2011). Antidepressant use during breastfeeding. *Current Women’s Health Reviews*.

[27] Berle JØ, Steen VM, Aamo TO, Breilid H, Zahlsen K, Spigset O (2004). Breastfeeding during maternal antidepressant treatment with serotonin reuptake inhibitors: infant exposure, clinical symptoms, and cytochrome P450 genotypes. *Journal of Clinical Psychiatry*.

[28] Ververs FFT, Voorbij HAM, Zwarts P (2009). Effect of cytochrome P450 2D6 genotype on maternal paroxetine plasma concentrations during pregnancy. *Clinical Pharmacokinetics*.

[29] Matsui DM (2012). Therapeutic drug monitoring in pregnancy. *Therapeutic Drug Monitoring*.

